# Hsa_circ_0072309 enhances autophagy and TMZ sensitivity in glioblastoma

**DOI:** 10.1111/cns.13821

**Published:** 2022-02-25

**Authors:** Fanen Yuan, Si Zhang, Qian Sun, Liguo Ye, Yang Xu, Zhou Xu, Gang Deng, Shenqi Zhang, Baohui Liu, Qianxue Chen

**Affiliations:** ^1^ Department of Neurosurgery Renmin Hospital of Wuhan University Wuhan China; ^2^ Central Laboratory Renmin Hospital of Wuhan University Wuhan China

**Keywords:** autophagy, circRNA, glioblastoma, temozolomide

## Abstract

**Aims:**

Circular RNAs have been reported to play key roles in the progression of various cancers, including gliomas. The present study was designed to investigate the role of hsa_circ_0072309 in autophagy and temozolomide (TMZ) sensitivity in glioblastoma (GBM).

**Methods:**

The effect of hsa_circ_0072309 on autophagy and TMZ sensitivity were examined by GFP‐RFP‐LC3, transmission electron microscopy(TEM), flow cytometry, Western blot, and immunofluorescence. The mechanism of hsa_circ_0072309 regulating p53 signaling pathway was analyzed using Western blot, IP, and rescue experiments.

**Results:**

Low hsa_circ_0072309 expression predicts poor prognosis for glioma patients. The regulation of hsa_circ_0072309 on autophagy and TMZ sensitivity depends on the status of p53. Hsa_circ_0072309 promoted autophagy by p53 signaling pathway and enhanced sensitivity of glioblastoma to temozolomide (TMZ) in p53 wild‐type GBM, but not in p53 mutant GBM. Hsa_circ_0072309 inhibits p53 ubiquitination and increases the stability of p53 protein in the context of p53 wild‐type. MiR‐100 mediates hsa_circ_0072309 regulating p53. P53 inhibitor or autophagy inhibitor could reverse the effect of hsa_circ_0072309 on TMZ sensitivity in p53 wild‐type GBM.

**Conclusions:**

This study revealed a function of hsa_circ_0072309 promoting autophagy by p53 signaling pathway and enhancing TMZ sensitivity. These findings demonstrated that hsa_circ_0072309 may be a potential and promising target in designing the treatment strategy for GBM.

## Graphical abstract

Yuan et al. describe a function of hsa_circ_0072309 promoting autophagy by p53 signaling pathway and enhancing TMZ sensitivity in p53 wild‐type GBM, but not in p53 mutant GBM. Hsa_circ_0072309 inhibits p53 ubiquitination and increases the stability of p53 protein. MiR‐100 mediates hsa_circ_0072309 regulating p53. P53 inhibitor or autophagy inhibitor could reverse the effect of hsa_circ_0072309 on TMZ sensitivity.

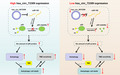

## 1 INTRODUCTION

Glioma is the most common type of central nervous tumor in adults and is associated with poor prognosis.^1^, ^2^ Glioblastoma (GBM) is the pathological type with the highest degree of malignancy in glioma. Despite the progress in surgical techniques, radiotherapy and chemotherapy, the prognosis of patients remains poor. Therefore, it is of great importance to explore the pathogenesis of glioblastoma and identify new therapeutic targets and treatment strategies.

CircRNAs are a class of single‐stranded covalently closed circular non‐coding RNAs with neither a 5′‐terminal nor 3′‐terminal poly A tail.^3^ Accumulating evidence revealed that circRNAs are associated with the development of many types of cancers. Gao et al.^4^ reported that circRNA circ_0001721 promotes the progression of osteosarcoma through miR‐372‐3p/MAPK7 Axis. Fang et al.^5^ reported that circ_0005075 stimulates the proliferation and metastasis of glioma via downregulating SIRT1. It is reported that hsa_circ_0008225 inhibits tumorigenesis of glioma via sponging miR‐890 and promoting ZMYND11 expression.^6^ However, the mechanisms and functions of circRNAs are not completely clear in gliomas.

Hsa_circ_0072309 is the splicing sequence of the exon of LIFR (chr5: 38523520‐38530768). It has been reported that the expression of hsa_circ_0072309 is decreased in breast cancer.^7^ Chen et al. found that hsa‐circ‐0072309 regulates the progression of renal cell carcinoma.^8^ Our previous study indicated that hsa_circ_0072309 is downregulated in glioblastoma and inhibits proliferation and invasion of glioblastoma.^8^ But the role of hsa_circ_0072309 on autophagy and TMZ sensitivity in glioblastoma remains unclear.

In the present study, we reported that low hsa_circ_0072309 expression predicts poor prognosis for glioma patients. The regulation of hsa_circ_0072309 on autophagy and TMZ sensitivity depends on the status of p53. Hsa_circ_0072309 promoted autophagy by p53 signaling pathway and enhanced sensitivity of glioblastoma to temozolomide (TMZ) in p53 wild‐type GBM, but not in p53 mutant GBM. Hsa_circ_0072309 inhibits p53 ubiquitination and increases the stability of p53 protein in the context of p53 wild‐type. MiR‐100 mediates hsa_circ_0072309 regulating p53. P53 inhibitor or autophagy inhibitor could reverse the effect of hsa_circ_0072309 on TMZ sensitivity in p53 wild‐type GBM.

## 2 MATERIALS AND METHODS

### 2.1 Cell lines and culture conditions

Glioblastoma cell lines U87, A172, and U251 were obtained from were obtained from the Cell Bank of the Shanghai Institute of Biochemistry and Cell Biology, Chinese Academy of Sciences (Shanghai, China). The p53 status of these cells: U87(p53 wild‐type), A172(p53 wild‐type), and U251(p53 R273H mutant). Cells were cultured in highglucose Dulbecco's Modified Eagle's Medium (Gibco, Thermo Fisher Scientific). All culture media were supplemented with 10% fetal bovine serum (Thermo Fisher Scientific), 1% penicillin/streptomycin. And the incubating temperature was 37°C, with 5% CO_2_.

### 2.2 Antibodies and reagents

Antibodies used in these experiments included the following: anti‐cleaved‐caspase3 (ab32042, Abcam), anti‐caspase3 (NB100‐56708SS, Novus), anti‐caspase9(ab32539, Abcam), anti‐BAX (50599‐2‐Ig, Proteintech), anti‐Bcl‐2 (GTX100064, GeneTex) anti‐GAPDH (#5174, Cell Signaling Technology), anti‐P62 (M162‐3, Medical Biological Laboratories), anti‐Beclin1 (11306‐1‐AP, Proteintech), anti‐LC3B (GB11124, Servicebio), anti‐ATG16L1(#8089, Cell Signaling Technology), anti‐ATG7(#8558, Cell Signaling Technology), anti‐p53(SC‐126, Santa Cruze), and anti‐ubiquitin(10201‐2‐AP, Proteintech).

Reagents used in these experiments included the following: autophagy inhibitor 3‐Methyladenine (3‐MA) (S2767, Selleck), p53 pathway inhibitor Pifithrin‐α (PFT‐α) (S2929, Selleck), temozolomide (TMZ) (S1237, Selleck), MG132 (S2619, Selleck), and cycloheximide (CHX)(HY‐12320, MCE).

### 2.3 CircRNA overexpression and knockdown

The construction of hsa_circ_0072309 overexpression and knockdown were described and verified in our previous study.^9^

### 2.4 Flow cytometric analysis

Apoptosis was determined by Annexin V‐PE/7‐AAD kit (Becton Dickinson, New Jersey). After being harvested and washed twice with PBS, cells were incubated with Annexin V‐PE/7‐AAD for 10 min and analyzed using a FACSCalibur flow cytometer (Becton Dickinson). Cells that are considered viable are PE Annexin V and 7‐AAD negative; cells that are in early apoptosis are PE Annexin V positive and 7‐AAD negative; and cells that are in late apoptosis or already dead are both PE Annexin V and 7‐AAD positive. The sum of upper right quadrant and low right quadrant were used for calculating total apoptosis rates and statistical analysis.

### 2.5 Western blot

Proteins were extracted by RIPA buffer (Beyotime) and protein concentration were determined by a BCA kit according to the manufacturer's instructions. Equal amount of protein was separated by SDS/PAGE and transferred to PVDF membranes (Millipore, Germany). After blocked in non‐fat milk for 1 h, the membranes were incubated in indicated primary antibodies overnight at 4 ^◦^C. Then membranes were washed three times with PBST, and incubated with Alex Fluor 680/790‐labeled secondary antibodies (LI‐COR Bioscience). Images were visualized using LI‐COR Odyssey Infrared Imaging System (LI‐COR Biosciences). The quantitative analysis of Western blots were presented in Figure S8.

### 2.6 Immunofluorescence

Cells were fixed and permeabilized using 4% paraformaldehyde and 0.5% Triton X‐100. And cells were blocked with 1% bovine serum albumin and incubated with primary antibodies. The cells were then incubated with Alexa fluor‐labeled secondary antibody (Antgene). All the images were captured with the fluorescence microscope and representative images were shown.

### 2.7 Immunohistochemistry

The tissues were embedded in paraffin after being fixed in 4% paraformaldehyde and cut into slices. The slices were treated in gradient hydration, followed by 3% H_2_O_2_ for 10 min and 1% BSA for 1 h. The samples were then incubated with primary antibodies. DAB staining was used to detect the signals, followed by hematoxylin counterstaining. Images were taken by Olympus BX51 microscope (Olympus).

### 2.8 Transmission Electron microscopy (TEM)

Cells were fixed with an electron fixation solution containing 2.5% glutaraldehyde, followed by post‐fixation in 1% osmic acid. Dehydration was performed in a graded series of ethanol. Then, the specimens were placed in capsules contained embedding medium and heated at 70°C for 9 h. The specimen sections were stained using uranyl acetate and alkaline lead citrate. The images were taken using a TEM (Hitachi HT7700).

### 2.9 Transfection of GFP‐RFP‐LC3 Adenoviruses

Cells were cultured in confocal dishes and then transfected with GFP‐RFP‐LC3 adenoviruses according to the manufacturer's instructions (Hanbio). Cells were observed under an Olympus FV1200 confocal microscope. Because GFP is sensitive to acidic conditions, when autophagosomes fuse with lysosomes, GFP fluorescence is quenched and only red fluorescence can be detected.^10^ The numbers of yellow puncta (GFP+RFP+) and red puncta (GFP‐RFP+) were counted for each cell, representing the autophagosomes and autolysosomes, respectively.

### 2.10 Intracranial xenograft model

The intracranial xenograft was constructed as our previous study described.^9^

This study was approved by the Institutional Animal Care and Use Committee at Renmin Hospital of Wuhan University.

### 2.11 Cell transfection

Ubiquitin plasmid with 6X His tag was constructed based on pcDNA3.1. miR‐100 mimic (Genepharma); and the negative control group were synthesized to alter the generation of miR‐100. Transfections were performed using Lipofectamine 3000 transfection reagent or Lipofectamine 2000 transfection reagent according to the manufacturer's instructions.

The sequence of hsa‐mir‐100‐5p mimics:

5′‐AACCCGUAGAUCCG11CUUGUG‐3′(Forward),

5′‐CAAGUUCGGAUCUACGGGUUUU‐3′(Reverse).

### 2.12 RNA extraction and quantitative real‐time PCR

RNA extraction and quantitative real‐time PCR were performed as described in our previous study.^9^ The primer sequences were as follows:

hsa_circ_0072309 5′‐ACACCGCTCAAATGTTATCTGG‐3′(Forward),

5′‐CAGGATGGTCGTTTCAAACATAC‐3′(Reverse);

GAPDH 5′‐GGAGCGAGATCCCTCCAAAAT‐3′(Forward),

5′‐GGCTGTTGTCATACTTCTCATGG‐3′(Reverse);

miR‐100 5′‐GAACCCGTAGATCCGAA‐3′(Forward),

5′‐CAGTGCGTGTCGTGGA‐3′(Reverse),

GTCGTATCCAGTGCGTGTCGTGGAGTCGGCAATTGCACTGGATACGACCACAAG(RT‐primer);

U6 5′‐CTCGCTTCGGCAGCACA‐3′(Forward),

5′‐AACGCTTCACGAATTTGCGT‐3′(Reverse);

### 2.13 circAtlas database

The circAtlas database contains an integrated resource of one million highly accurate circular RNAs from 1070 vertebrate transcriptomes.^11^ From circAtlas, we analyzed the expression pattern of hsa_circ_0072309 and its host gene(LIFR) in a variety of human tissues, as well as its junction ration.

### 2.14 The Chinese Glioma Genome Atlas (CGGA) database

The microRNA‐seq data and indicated information of glioma samples were obtained from The Chinese Glioma Genome Atlas (CGGA) database (https://www.cgga.org.cn/download.jsp). The data set used in CGGA database is “microRNA_array_198.” The microRNA expression analysis and survival analysis were performed using the data from CGGA.

### 2.15 Clinical samples

All clinical tissues were obtained from the Department of Neurosurgery, Renmin Hospital of Wuhan University, Wuhan, China. The written informed consent was signed by the patients or family members. The Institutional Ethics Committee of the Faculty of Medicine at Renmin Hospital of Wuhan University has approved this project.

### 2.16 Subcutaneous glioma xenograft model

Mice were randomly divided into four groups: U87‐PCDH+TMZ group, U87‐72309+TMZ group, U87‐PCDH+TMZ+3‐MA group, and U87‐72309+TMZ+3‐MA group. Cells were subcutaneously inoculated to the ventral side of nude mice. TMZ (temozolomide) were administrated every 3 days at a 10 mg/kg dose; 3‐MA were administrated every 3 days at 10 mg/kg. After subcutaneous implantation, the tumor volume was monitored every 5 days. Recorded dynamic changes in the size of subcutaneous xenografts (length × width ^2^/ 2).

The animal data reporting has followed the ARRIVE guidelines.^12^

### 2.17 Statistical analysis

The results are representative of the three independent experiments. The normality of the data distribution was analyzed by the Shapiro–Wilk test. The experimental results are expressed as the means  ± SEM. Statistical comparisons of data were performed using Student's *t*‐test or one‐way analysis of variance. Kaplan–Meier curve was performed for survival analysis and *p* value was calculated by log rank test. *p* < 0.05 indicated that the differences were statistically significant. Data were analyzed with GraphPad Prism 5 software.

## 3 RESULTS

### 3.1 Low hsa_circ_0072309 expression predicts poor prognosis for glioma patients

**FIGURE 1:**
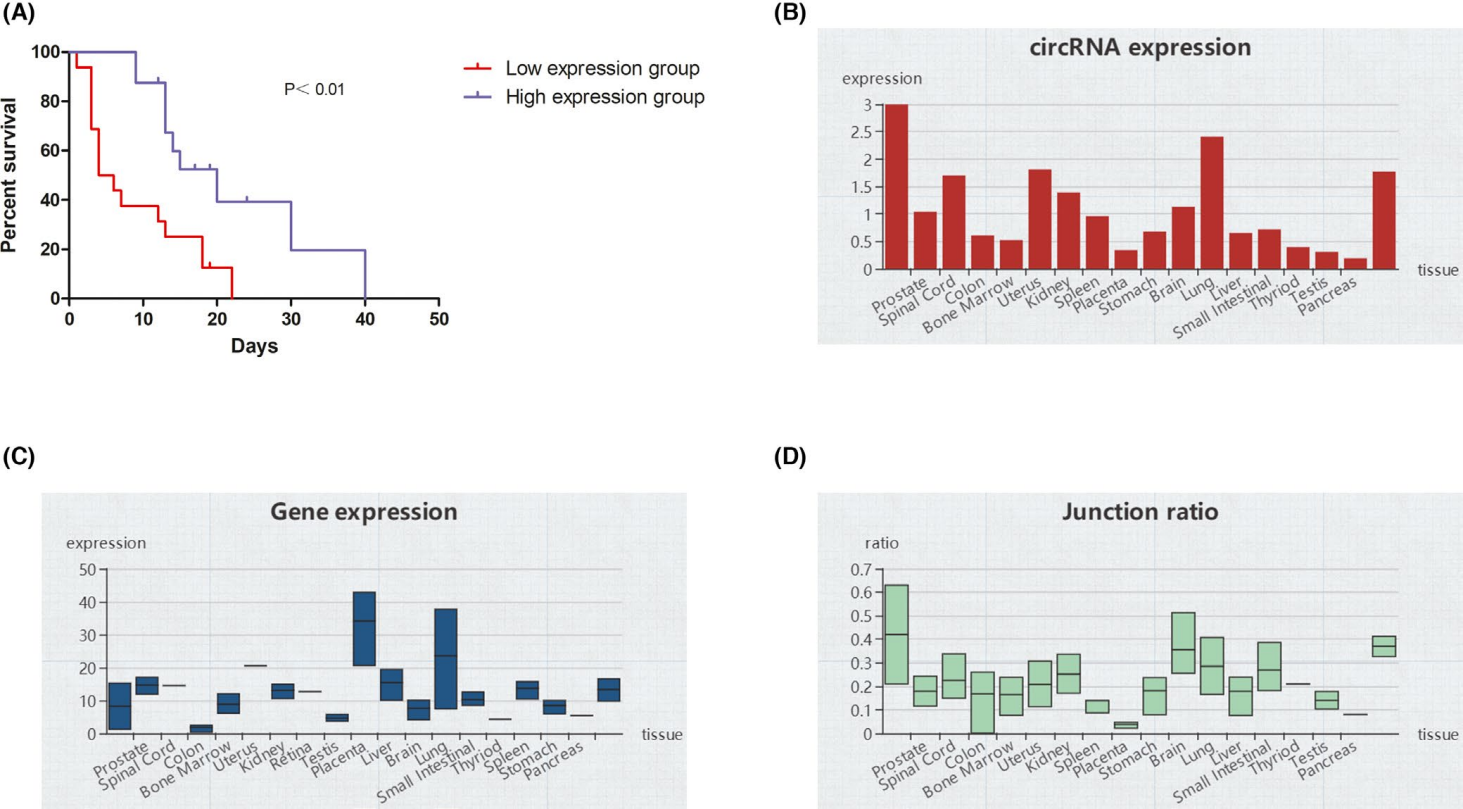
Low hsa_circ_0072309 expression predicts poor prognosis for glioma patients. (A) Kaplan–Meier survival analysis for hsa_circ_0072309 expression in glioma patients (*n* = 32). Low‐expression group: *n =* 16. High‐expression group: *n =* 16. P value was calculated by log rank test. (B) The expression pattern of hsa_circ_0072309 in a variety of human tissues from circAtlas database. (C) The expression pattern of LIFR (the host gene of hsa_circ_0072309) in a variety of human tissues from circAtlas database. (D)The junction ratio of hsa_circ_0072309 in a variety of human tissues from circAtlas database

Our previous study has demonstrated that the expression of hsa_circ_0072309 is downregulated in GBM patients.^9^ In the present study, we tried to explore the prognostic value of hsa_circ_0072309 in gliomas. All glioma patients were divided into two groups according to the expression level of hsa_circ_0072309 in 50% cut‐off point. Kaplan–Meier analysis was performed. The results indicated that glioma patients with low hsa_circ_0072309 expression had worse survival than those with high hsa_circ_0072309 expression (Figure 1A). From circAtlas database (http://circatlas.biols.ac.cn/)^11^, we analyzed the expression pattern of hsa_circ_0072309 and its host gene(LIFR) in a variety of human tissues (Figure 1B,C), as well as its junction ration (Figure 1D). This result showed us a brief expression pattern of hsa_circ_0072309 and its host gene(LIFR) in a variety of human tissues, as well as the junction ratio.

### 3.2 Hsa_circ_0072309 promotes autophagy in p53 wild‐type GBM

**FIGURE 2:**
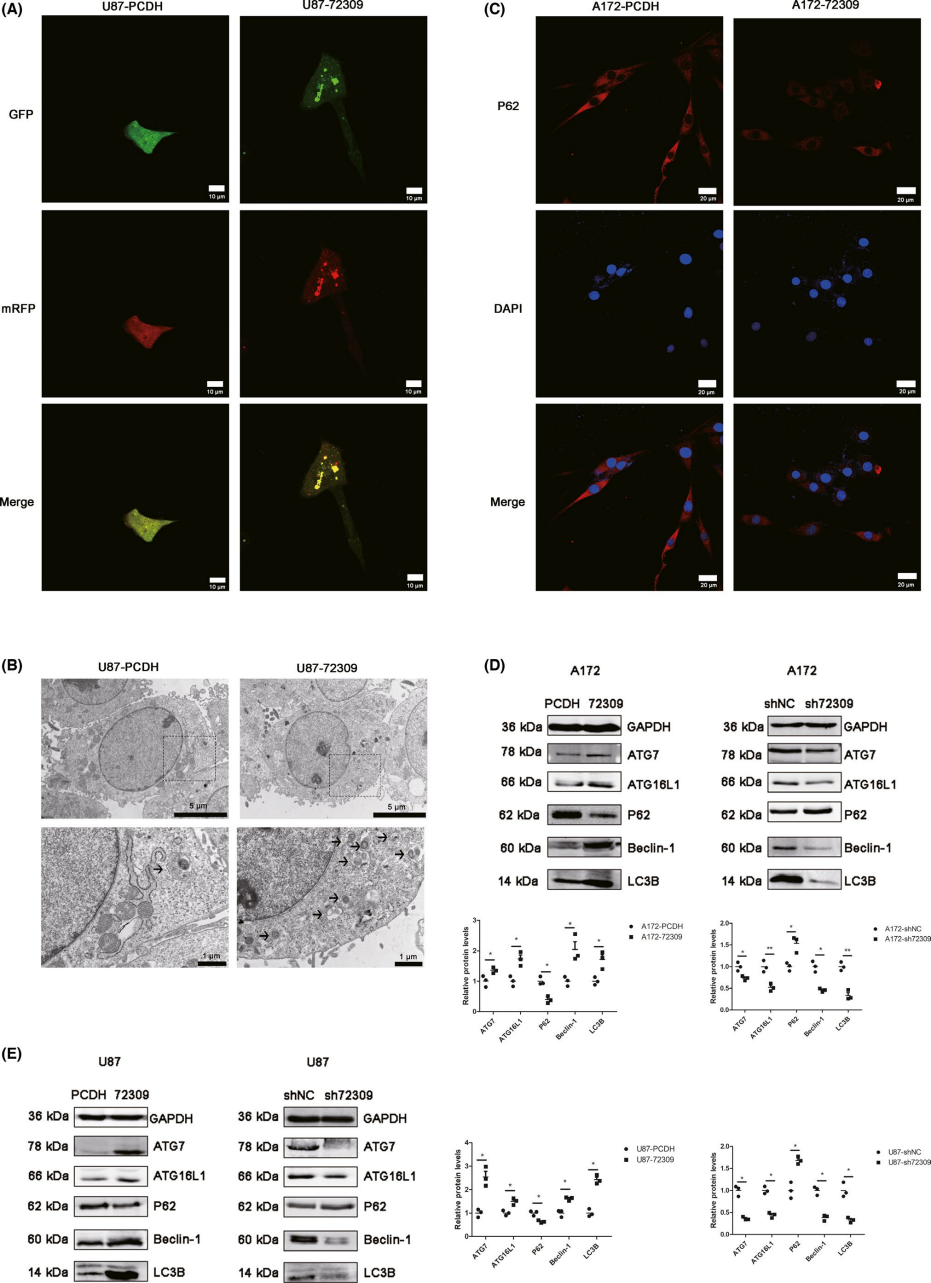
Hsa_circ_0072309 promotes autophagy in p53 wild‐type GBM. (A) Autophagic flux was determined by diploid adenovirus (mRFP‐GFP‐LC3). Representative images of fluorescent LC3 puncta in U87‐PCDH and U87‐72309 cells are shown. Red dots represent autolysosomes and yellow dots autophagosomes, scale bars =10 μm. (B) U87‐PCDH and U87‐72309 cells were analyzed by transmission electron microscopy. Black arrow indicates autophagosome. Scale bars represent 5 μm and 1 μm. (C) Immunofluorescence was used to determine the effect of hsa_circ_0072309 overexpression on p62 in A172 cells. Scale bars =20 μm. (D) Western blot was used to determine the effect of hsa_circ_0072309 overexpression or depletion on the expression of ATG7, ATG16L1, Beclin‐1, P62, and LC3B in A172 cells. (E) Western blot was used to determine the effect of hsa_circ_0072309 overexpression or depletion on the expression of ATG7, ATG16L1, Beclin‐1, P62, and LC3B in U87 cells. A172/U87‐PCDH: the control group. A172/U87‐72309: the hsa_circ_0072309 overexpressed group. A172/U87‐shNC: the negative control group. A172/U87‐sh72309: the hsa_circ_0072309 knockdown group

The stable overexpression or knockdown of hsa_circ_0072309 were constructed as our previous study described.^9^ The overexpression or knock‐down efficiency were validated by qPCR (Figure S1). The p53 statuses of U87 and A172 cells are wild‐type, while U251 cells are p53 mutant (p53 R273h mutant). At first, the phenotype experiments were performed in p53 wild‐type GBM. We use multiple methods to determine the levels of autophagy. A diploid adenovirus (mRFP‐GFP‐LC3) was performed to indicate autophagic flux: red dots represent autolysosomes and yellow dots autophagosomes. The results indicated that hsa_circ_0072309 overexpression lead to enhanced autophagic flux in p53 wild‐type GBM cells (Figure 2A). Transmission electron microscopy showed that the number of autophagic vacuoles increased in p53 wild‐type GBM cells with hsa_circ_0072309 overexpression (Figure 2B). Immunofluorescence was performed to examine the expression of SQSTM1/P62, and the results indicated that the expression of p62 decreased in A172 cells with hsa_circ_0072309 overexpression (Figure 2C) and in U87 cells with hsa_circ_0072309 overexpression (Figure S2). Western blot was performed to detect the expression of ATG7, ATG16L1, Beclin‐1, P62, and LC3B. The results showed that hsa_circ_0072309 overexpression lead to increased expression of ATG7, ATG16L1, Beclin‐1, and LC3B, and decreased expression of P62 in A172 cells; while knocking down hsa_circ_0072309 lead to decreased expression of ATG7, ATG16L1, Beclin‐1, LC3B, and increased expression of p62 in A172 cells (Figure 2D). The similar results were confirmed in U87 cells (Figure 2E). These results indicate that hsa_circ_0072309 promotes autophagy in p53 wild‐type GBM.

### 3.3 Hsa_circ_0072309 enhances TMZ sensitivity in p53 wild‐type GBM

**FIGURE 3:**
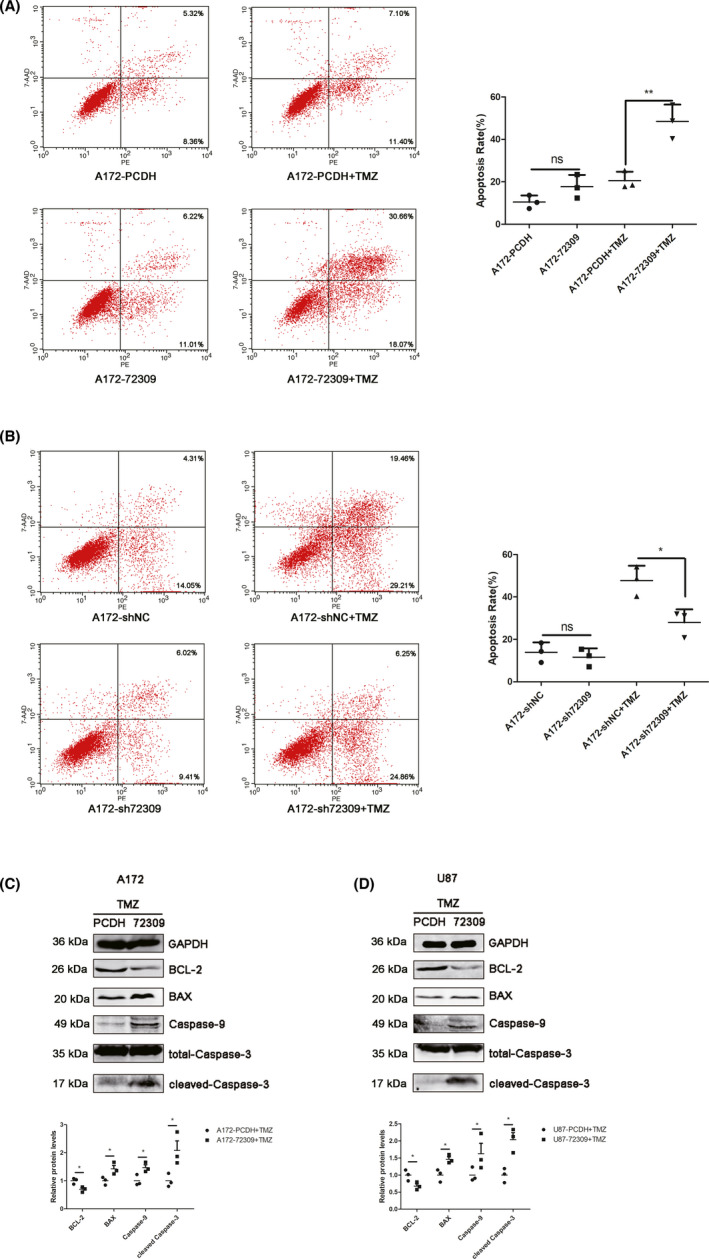
Hsa_circ_0072309 enhances TMZ sensitivity in p53 wild‐type GBM. (A) Annexin V‐PE/7‐AAD staining and flow cytometric analysis were performed to detect the effect of hsa_circ_0072309 overexpression on TMZ‐induced apoptosis in A172 cells. *p < 0.05. (B) Annexin V‐PE/7‐AAD staining and flow cytometric analysis were performed to detect the effect of hsa_circ_0072309 depletion on TMZ‐induced apoptosis in A172 cells. *p < 0.05. (C) Western blot was used to determine the effect of hsa_circ_0072309 overexpression on the expression of Bax, caspase‐9, cleaved‐caspase‐3, total‐caspase‐3, and Bcl‐2 in A172 cells treated with TMZ. (D) Western blot was used to determine the effect of hsa_circ_0072309 overexpression on the expression of Bax, caspase‐9, cleaved‐caspase‐3, total‐caspase‐3, and Bcl‐2 in U87 treated with TMZ cells. The graphs are representative of three independent experiments with similar results. A172/U87‐PCDH: the control group. A172/U87‐72309: the hsa_circ_0072309 overexpressed group. A172‐shNC: the negative control group. A172‐sh72309: the hsa_circ_0072309 knockdown group

To investigate whether hsa_circ_0072309 affects TMZ sensitivity, we performed flow cytometry with Annexin V‐PE/7‐AAD staining to determine the apoptosis levels of p53 wild‐type GBM cells treated with TMZ. Annexin V‐PE/7‐AAD staining demonstrated that, in p53 wild‐type GBM cells, hsa_circ_0072309 overexpression promotes apoptosis induced by TMZ (Figure 3A), while hsa_circ_0072309 depletion attenuates apoptosis induced by TMZ (Figure 3B). Western blots showed that hsa_circ_0072309 overexpression lead to increased expression of Bax, caspase‐9, cleaved‐caspase‐3, and decreased expression of Bcl‐2 in p53 wild‐type GBM cells (Figure 3C, D). These data indicate that hsa_circ_0072309 enhances TMZ sensitivity in p53 wild‐type GBM.

### 3.4 Hsa_circ_0072309 had no significant effect on autophagy, TMZ sensitivity, and p53 expression in p53 mutant GBM

**FIGURE 4:**
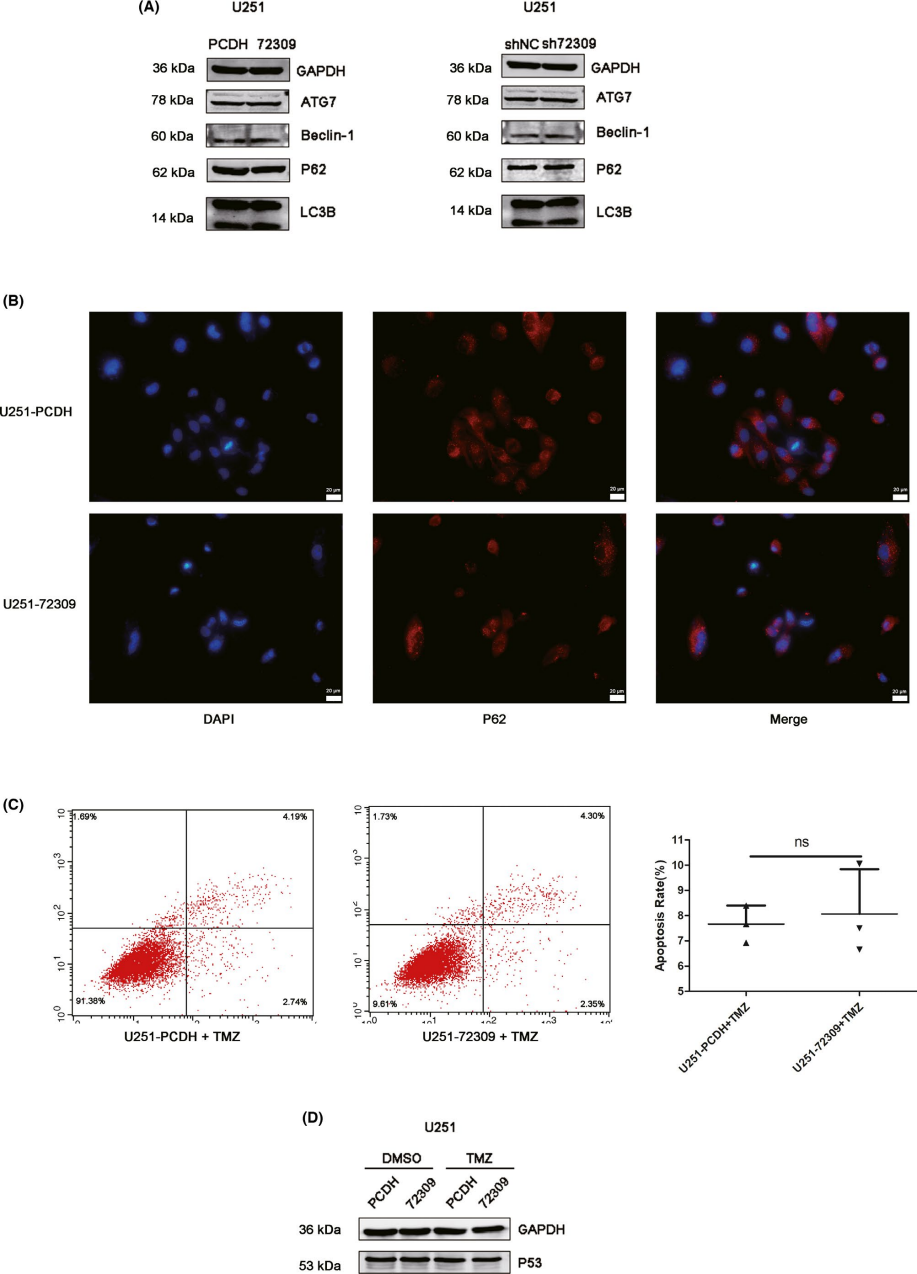
Hsa_circ_0072309 had no significant effect on autophagy, TMZ sensitivity, and p53 expression in p53 mutant GBM. (A) Western blot was used to determine the effect of hsa_circ_0072309 overexpression or depletion on the expression of ATG7, Beclin‐1, P62, and LC3B in U251 cells. (B) Immunofluorescence was used to determine the effect of hsa_circ_0072309 overexpression on p62 in U251 cells. scale bars =20 μm. (C) Annexin V‐PE/7‐AAD staining and flow cytometric analysis were performed when the hsa_circ_0072309 overexpressed group and the control group were treated with TMZ; ns, not significant. U251‐PCDH: the control group. U251‐72309: the hsa_circ_0072309 overexpressed group. U251‐shNC: the negative control group. U251‐sh72309: the hsa_circ_0072309 knockdown group. (D) Western blot was performed to determine the effect of hsa_circ_0072309 overexpression on the expression of p53 in U251 cells treated with or without TMZ

Phenotype experiments were also performed in p53 mutant GBM cells. The p53 status of U251 cells are mutant (p53 R273h mutant). Western blots showed that hsa_circ_0072309 overexpression or depletion had no significant effect on the expression of autophagic markers including ATG7, p62, LC3B, and Beclin‐1 in U251 cells (Figure 4A). Immunofluorescence was performed to examined the expression of p62, and results indicated that hsa_circ_0072309 overexpression had no significant effect on p62 expression in U251 cells (Figure 4B). Also, hsa_circ_0072309 overexpression had no significant impact on autophagy flux in U251 cells using GFP‐RFP‐LC3 adenoviruses transfection (Figure S3). Annexin V‐PE/7‐AAD staining demonstrated that, in p53 mutant GBM cells, hsa_circ_0072309 overexpression had no significant effect on apoptosis induced by TMZ (Figure 4C). Hsa_circ_0072309 depletion had no significant effect on TMZ‐induced apoptosis in U251 cells (Figure S4). Moreover, hsa_circ_0072309 overexpression did not affect p53 expression in U251 cells treated with TMZ or not (Figure 4D). These results demonstrated that hsa_circ_0072309 had no significant effect on autophagy, TMZ sensitivity, and p53 expression in p53 mutant GBM.

### 3.5 Hsa_circ_0072309 inhibits p53 ubiquitination and increases the stability of p53 protein via miR‐100 in the context of wild‐type p53

**FIGURE 5:**
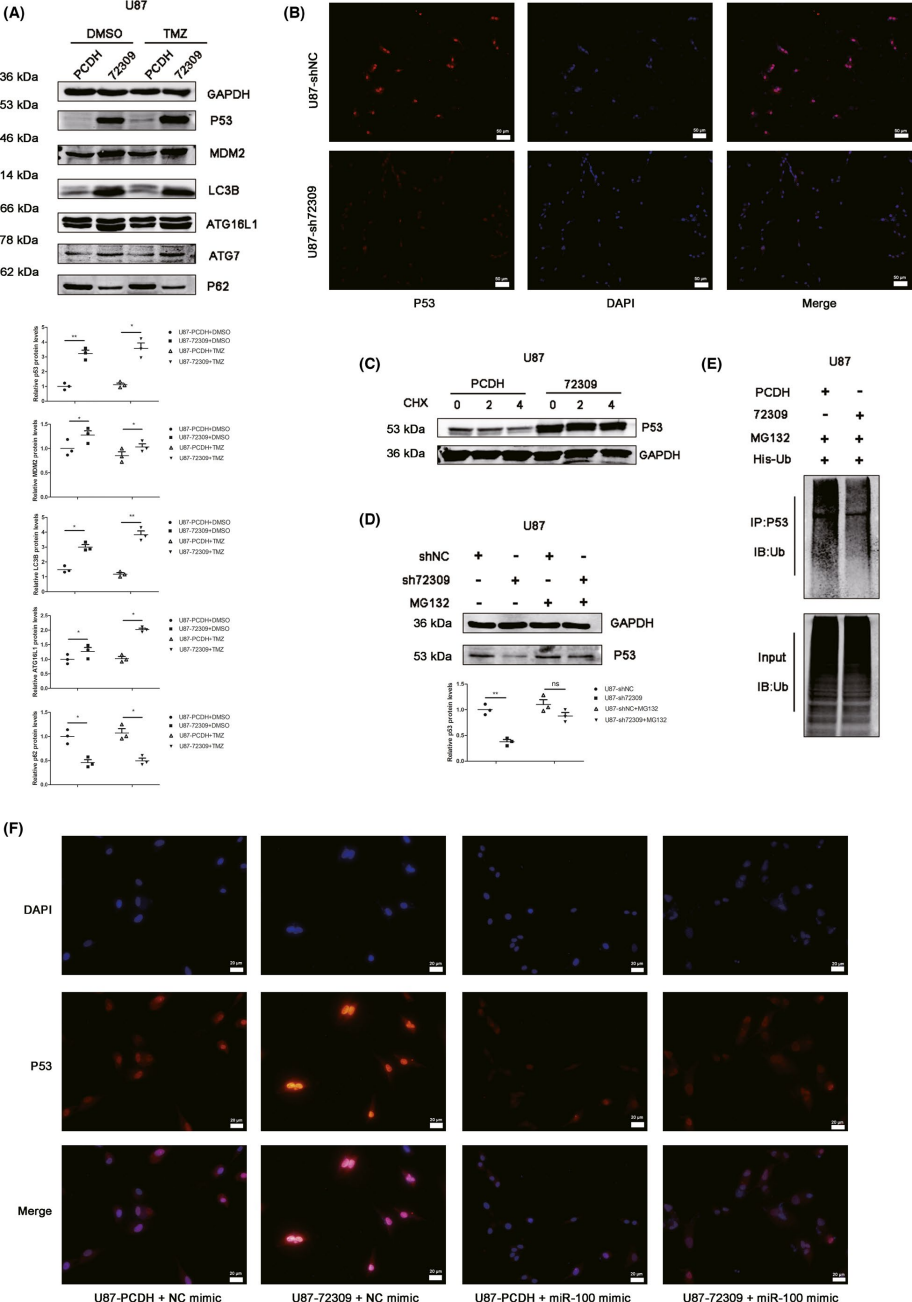
Hsa_circ_0072309 increases the stability of p53 protein by inhibiting p53 ubiquitination via miR‐100 in the context of wild‐type p53. (A) Western blot was performed to determine the effect of hsa_circ_0072309 overexpression on the expression of p53, MDM2, ATG16L1, ATG7, LC3B, and p62 in U87 cells treated with or without TMZ. (B) Immunofluorescence was used to determine the effect of hsa_circ_0072309 depletion on p53 in U87 cells. scale bars =50 μm. (C) A cycloheximide (CHX)‐chase experiment was performed to compare the rate of p53 degradation between the hsa_circ_0072309 overexpressed group and the control group. (D) Western blot was used to detect the level of p53 when the hsa_circ_0072309 depletion group and the control group were treated with MG132 or not. (E) U87‐PCDH and U87‐72309 cells were transfected with His‐ubiquitin, followed by treatment of MG132. The proteins were immunoprecipitated using anti‐p53 antibody, followed by immunoblotting with anti‐ubiquitin antibodies. (F) Immunofluorescence was performed to determine the expression of p53 in U87‐PCDH and U87‐72309 cells transfected with miR‐100 mimic or NC mimic. The graphs are representative of three independent experiments with similar results. U87‐PCDH: the control group. U87‐72309: the hsa_circ_0072309 overexpressed group. U87‐shNC: the negative control group. U87‐sh72309: the hsa_circ_0072309 knockdown group

P53 is considered to be an important regulator in autophagy and chemoresistance.^13^, ^14^ Our phenotype experiments had shown that hsa_circ_0072309 promotes autophagy and enhances TMZ sensitivity in p53 wild‐type GBM, but not in p53 mutant GBM. It indicates the key role of p53 in hsa_circ_0072309‐mediated regulation. Thus, we aim to explore whether hsa_circ_0072309 regulates p53 in p53 wild‐type GBM. Western blot showed that hsa_circ_0072309 overexpression lead to increased expression of p53, MDM2, ATG16L1, ATG7, LC3B, and decreased expression of p62 (Figure 5A). Immunofluorescence also confirmed that hsa_circ_0072309 depletion caused a decrease of p53 expression (Figure 5B). In a cycloheximide (CHX) pulse‐chase assay, we found that hsa_circ_0072309 overexpression stabilized p53 protein (Figure 5C). In the presence of the proteasome inhibitor (MG132), hsa_circ_0072309 depletion did not further decrease p53 protein expression (Figure 5D). Then, we examined p53 ubiquitination by immunoprecipitation of p53 followed by immunoblot analysis of ubiquitinated proteins. We found that the amount of p53 ubiquitinated protein was decreased after hsa_circ_0072309 overexpression (Figure 5E). These results indicate that hsa_circ_0072309 increases the stability of p53 protein by inhibiting p53 ubiquitination in the context of wild‐type p53.

Then, we try to clarify how hsa_circ_0072309 inhibits p53 ubiquitination and increases the stability of p53 protein. circRNA could exert its biological function by acting as a competing endogenous RNA (ceRNA). Several studies demonstrated that hsa‐circ‐0072309 plays anti‐tumor roles by sponging miR‐100.^8^, ^15^ Moreover, miR‐100 antagonism inhibited ubiquitin‐mediated p53 protein degradation by activating RNF144B, an E3 ubiquitination ligase.^16^ Thus, we speculated that hsa_circ_0072309 may inhibit p53 protein degradation via sponging miR‐100. From the Chinese Glioma Genome Atlas (CGGA) database, the expression pattern and survival analysis were performed. The results showed that miR‐100 expression was elevated as with the increase of glioma pathological grades (Figure S5A). Glioma patients with high miR‐100 expression had poorer survival time than those with low miR‐100 expression (Figure S5B), despite whether it is primary or recurrent. Immunofluorescence was performed to determine the expression of p53 in U87‐PCDH and U87‐72309 cells, and miR‐100 mimic was used for the rescue experiment. The results demonstrated that hsa_circ_0072309 overexpression lead to increased p53 expression, and miR‐100 mimic could reverse that effect (Figure 5F), indicating miR‐100 mediates hsa_circ_0072309 regulating p53. Western blot was used to detect the protein level of p53, ATG16L1, and p62 in U87‐PCDH group, U87‐72309 group, U87‐PCDH+miR‐100 mimic group, and U87‐72309+miR‐100 mimic group. The results indicated that miR‐100 mimic could reverse the effect of hsa_circ_0072309 overexpression on the expression of p53, ATG16L1, and p62 (Figure S6).

### 3.6 Hsa_circ_0072309 promotes autophagy by p53 signaling pathway

**FIGURE 6:**
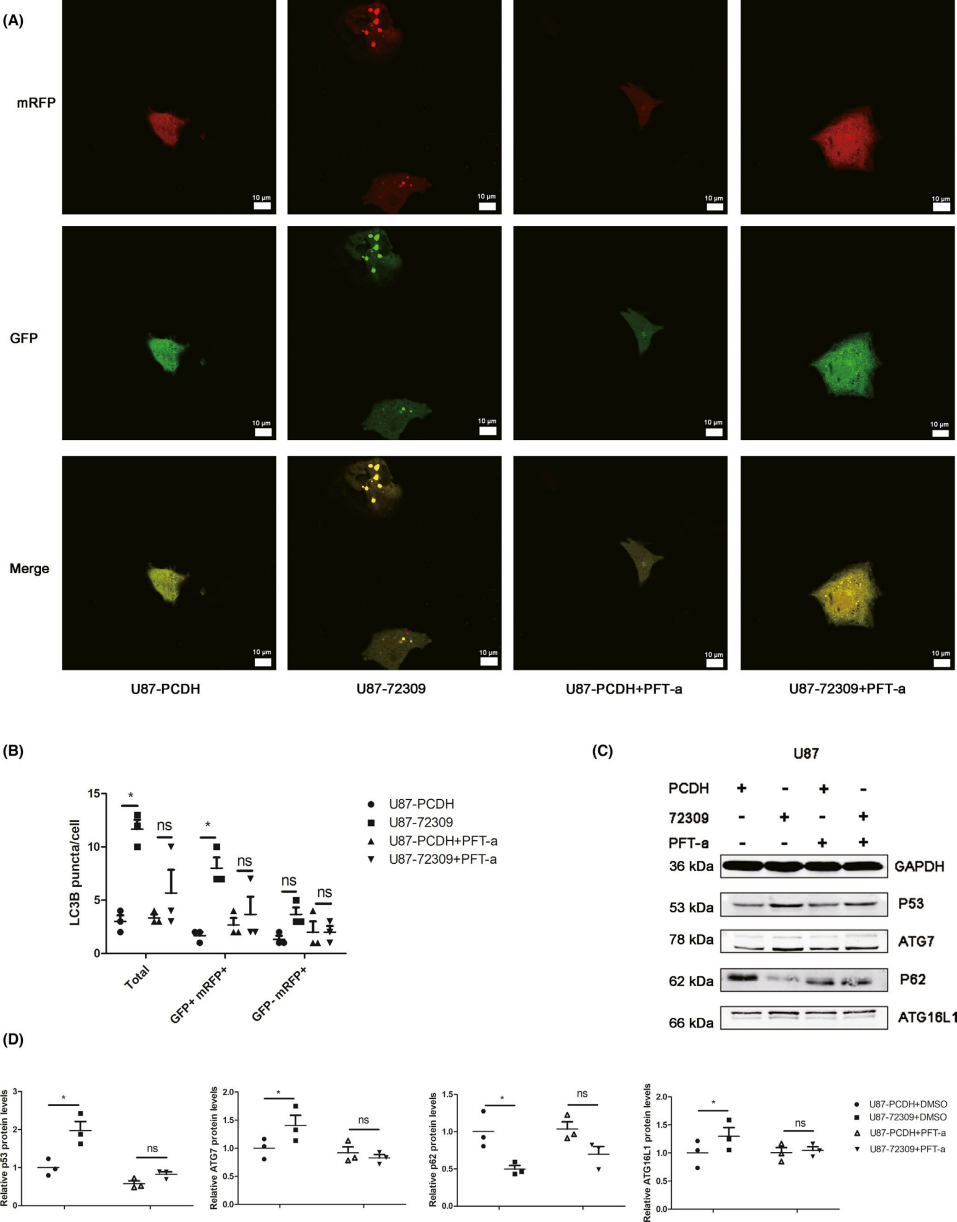
Hsa_circ_0072309 promotes autophagy by p53 signaling pathway. (A) The hsa_circ_0072309 overexpressed cells and the control cells were treated with pifithrin‐α(PFT‐α) or not. Autophagic flux was determined by diploid adenovirus (mRFP‐GFP‐LC3). Representative images of fluorescent LC3 puncta are shown. Red dots represent autolysosomes and yellow dots autophagosomes, scale bars =10 μm. (B) The statistics of LC3 puncta in Figure 6A. (C) Western blot was used to detect the protein level of ATG16L1, ATG7, and p62 when the hsa_circ_0072309 overexpressed cells and the control cells were treated with pifithrin‐αor not. The graphs are representative of three independent experiments with similar results. U87‐PCDH: the control group. U87‐72309: the hsa_circ_0072309 overexpressed group. (D) The quantification of Figure 6C

In order to verify whether hsa_circ_0072309 promotes autophagy via p53 signaling pathway, we use pifithrin‐α, a p53 pathway inhibitor, to perform rescue experiment. In the experiment using mRFP‐GFP‐LC3 transfection to assess autophagic flux, we found that pifithrin‐α rescued the effect of hsa_circ_0072309 overexpression on autophagic flux (Figure 6A,B). Western blot was used to detect the protein level of ATG16L1, ATG7, and p62 when the hsa_circ_0072309 overexpressed cells (U87‐72309) and the control cells (U87‐PCDH) were treated with pifithrin‐αor not. The results indicated that pifithrin‐α reversed the effect of hsa_circ_0072309 overexpression on the expression of ATG16L1, ATG7, and p62 (Figure 6C,D). These results indicate that p53 signaling pathway is required for the autophagy mediated by hsa_circ_0072309.

### 3.7 Pifithrin‐α or 3‐MA could reverse the effect of hsa_circ_0072309 on TMZ sensitivity in glioblastoma

**FIGURE 7:**
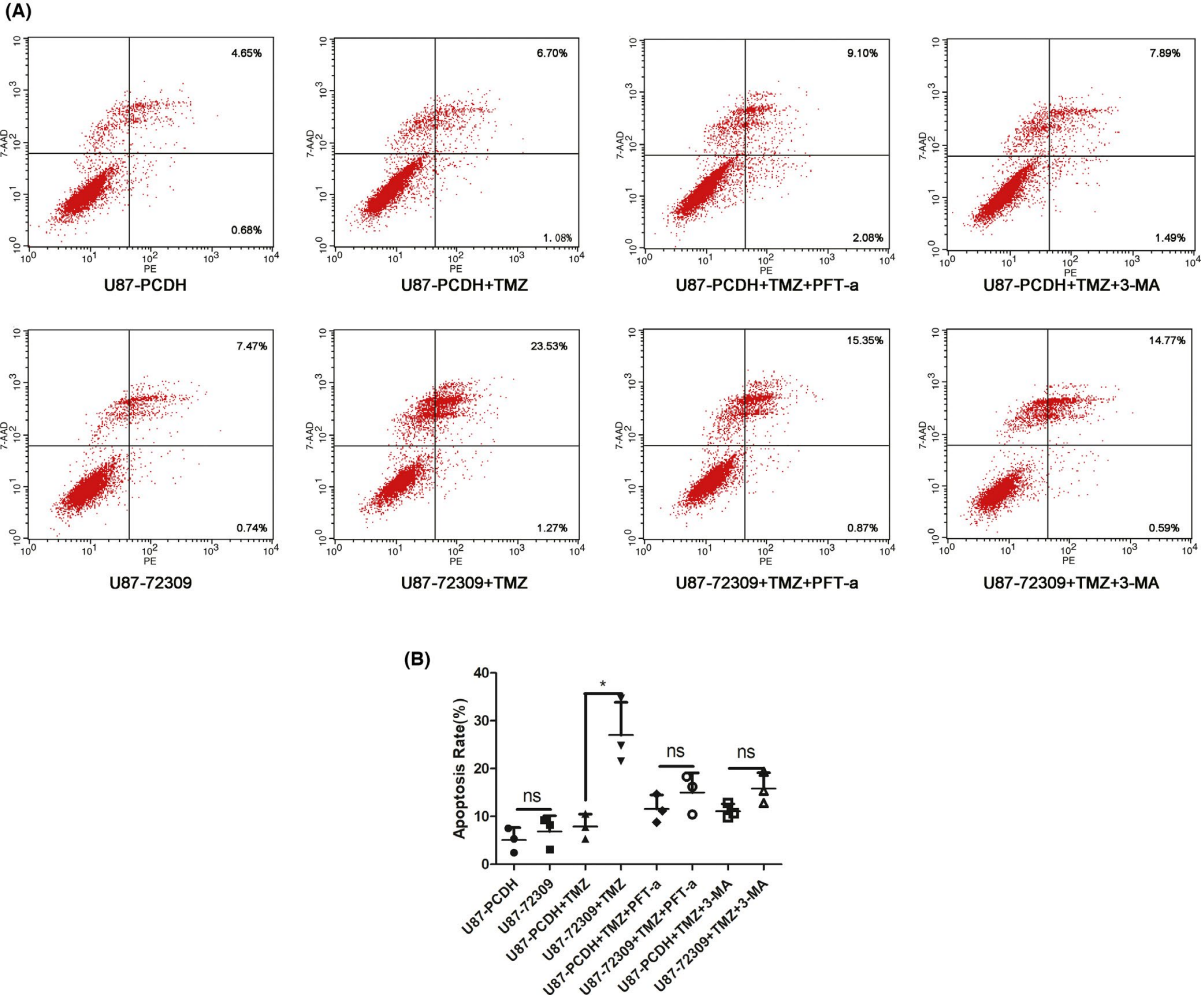
Pifithrin‐α or 3‐MA could reverse the effect of hsa_circ_0072309 on TMZ sensitivity. (A) Annexin V‐PE/7‐AAD staining and flow cytometric analysis were performed when the hsa_circ_0072309 overexpressed group and the control group were treated with TMZ or TMZ+3‐MA or TMZ+Pft‐αor not. *p < 0.05. (B) The statistics of apoptosis rates in Figure 7A. The graphs are representative of three independent experiments with similar results. U87‐PCDH: the control group. U87‐72309: the hsa_circ_0072309 overexpressed group

To explore the role of p53 and autophagy on TMZ sensitivity mediated by hsa_circ_0072309, we employed pifithrin‐α (p53 inhibitor) and 3‐MA(autophagy inhibitor) to perform rescue experiment. Flow cytometry with Annexin V‐PE/7‐AAD staining indicated that hsa_circ_0072309 overexpression enhanced TMZ‐induced apoptosis in U87 cells, but the treatment of pifithrin‐α or 3‐MA could attenuate that effect of hsa_circ_0072309 (Figure 7A, B). These results showed that p53 inhibitor or autophagy inhibitor could reverse the effect of hsa_circ_0072309 on sensitivity of glioblastoma to TMZ, indicating that hsa_circ_0072309 mediated chemoresistance to TMZ is p53‐dependent and autophagy‐related.

### 3.8 Hsa_circ_0072309 promotes the autophagy of glioblastoma in vivo

**FIGURE 8:**
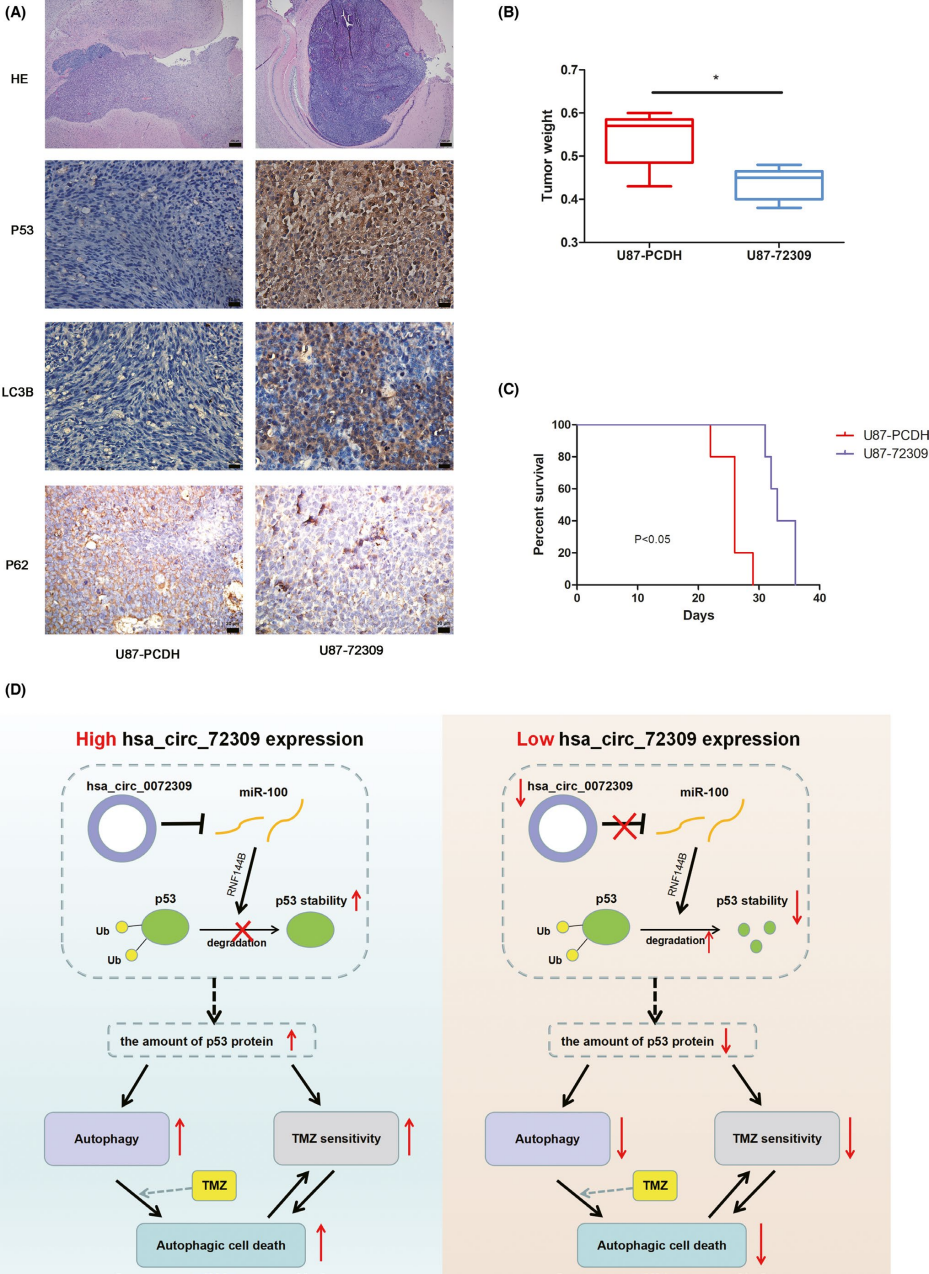
Hsa_circ_0072309 promotes the autophagy of glioblastoma in vivo. (A) HE and IHC analyses of p53, p62, and LC3B in orthotopic tumor sections. (B) The weight of tumors was analyzed. *p < 0.05. (C) Mouse survival is shown by Kaplan–Meier curves. P value was calculated by log rank test. (D) Mechanistic model for hsa_circ_0072309 regulating autophagy and TMZ sensitivity. In the context of p53 wild type, hsa_circ_0072309 may inhibits p53 ubiquitination and prevents p53 degradation via sponging miR‐100. With the amount of p53 increased, autophagic cell death is activated and the sensitivity to TMZ is enhanced

To investigate the role of hsa_circ_0072309 on autophagy in vivo, we performed.

an intracranial xenograft model.^9^ Immunohistochemistry staining indicated that the level of p53 and LC3B were higher in hsa_circ_0072309 overexpressed group (U87− 72309) than which in the control group (U87‐PCDH) (Figure 8A), while the level of p62 was lower in hsa_circ_0072309 overexpressed group (U87‐72309) than which in the control group (U87‐PCDH) (Figure 8A). The weights of the tumors in the hsa_circ_0072309 overexpression group were lower than the control group (Figure 8B). Kaplan–Meier curves showed that hsa_circ_0072309 overexpression group mice survived significantly longer than the control group (Figure 8C). These results indicated that hsa_circ_0072309 promotes autophagy of glioblastoma in vivo.

Moreover, subcutaneous glioma xenograft model was performed to validate the role of hsa_circ_0072309 on TMZ sensitivity. The results (Figure S7A) indicated that hsa_circ_0072309 overexpression enhanced TMZ sensitivity, and this change may be partially attenuated by 3‐MA. Expression of hsa_circ_0072309 and miR‐100 were measured by qPCR. The results indicated that the expression of miR‐100 was significant lower in hsa_circ_0072309 overexpressed group than the control group (Figure S7B).

## 4 DISCUSSION

Accumulating evidence indicated that circRNA plays an important role in glioma.^17^, ^18^ Our study identified hsa_circ_0072309 as a regulator of autophagy and TMZ sensitivity depending on the status of p53. Low hsa_circ_0072309 expression predicts poor prognosis for glioma patients. The regulation of hsa_circ_0072309 on autophagy and TMZ sensitivity depends on the status of p53. Hsa_circ_0072309 promoted autophagy by p53 signaling pathway and enhanced sensitivity of glioblastoma to temozolomide (TMZ) in p53 wild‐type GBM, but not in p53 mutant GBM. Hsa_circ_0072309 inhibits p53 ubiquitination and increases the stability of p53 protein in the context of p53 wild‐type. P53 inhibitor or autophagy inhibitor could reverse the effect of hsa_circ_0072309 on TMZ sensitivity in p53 wild‐type GBM.

CircRNAs are novel RNA molecules with multiple biological functions. Our studies showed that hsa_circ_0072309 increased the stability of p53 protein and p53 signaling pathway is required for the autophagy mediated by hsa_circ_0072309. But what is the exact molecular mechanism by which hsa_circ_0072309 increased the stability of p53 protein? circRNA could exert its biological function by acting as a competing endogenous RNA (ceRNA). Chen et al. demonstrated that hsa‐circ‐0072309 plays anti‐tumor roles by sponging miR‐100.^8^ Zhao et al.^15^ also confirmed that hsa_circ_0072309 could directly bind to miR‐100. Notably, miR‐100 antagonism inhibited ubiquitin‐mediated p53 protein degradation by activating RNF144B, an E3 ubiquitination ligase.^15^ Based on the literature and our results, we propose a hypothesis that hsa_circ_0072309 may inhibit p53 protein degradation via sponging miR‐100 (Figure 8D). In brief, with the context of p53 wild type, hsa_circ_0072309 may inhibits p53 ubiquitination and prevents p53 degradation via sponging miR‐100. With the amount of p53 increased, autophagic cell death is activated and the sensitivity to TMZ is enhanced. However, this hypothesis needs more experiments to clarify the relationship and exact regulatory mechanism of hsa_circ_0072309/miR‐100/p53.

Temozolomide (TMZ) is the key drug in GBM chemotherapy. Chemoresistance to TMZ is a major challenge in the treatment of glioblastoma. It was reported that TMZ could induce autophagy and apoptosis in GBM cells.^19^ Autophagy plays an important role in TMZ resistance in GBM. Autophagy can promote or attenuate tumor resistance, depending on whether it is cytoprotective or cytotoxic. On one hand, TMZ‐induced autophagy seems to have a cytoprotective role. It was reported that autophagy inhibitor enhanced the cytotoxicity of TMZ for malignant gliomas.^20^ On the other hand, autophagy‐associated cell death was reported be required for the cytotoxicity of TMZ and radiotherapy.^21^ Kanzawa et al.^20^ found that additional treatment of TMZ‐treated GBM cells with Bafilomycin A1, which inhibits autophagy at the late stage, resulted in increased TMZ cytotoxicity. While 3‐methyladenine (3‐MA), which inhibits autophagy at the early stage, resulted in decreased TMZ cytotoxicity.^20^ Based on these researches, autophagy‐associated cell death might constitute a possible adjuvant therapeutic strategy to enhance conventional GBM treatments. In our study, we found that 3‐MA could reverse the effect of hsa_circ_0072309 on sensitivity of glioblastoma to TMZ. Further studies are needed to figure out how different autophagy inhibitors or regulators play different roles in different stages of TMZ‐related autophagy to exert different influence.

P53 can play dual role in autophagy. It is usually believed that nuclear p53 induces autophagy by transcriptional effects, whereas cytoplasmic p53 acts as an inhibitor of autophagy.^22^ Many autophagy‐related genes are regulated by p53, including Ulk1 and Atg7.^23^ This p53‐induced autophagy revealed stimulation of apoptosis in response to DNA damage.^23^ Besides the transcriptional regulation, non‐transcriptional mechanisms by which cytoplasmic p53 can suppress autophagy have also been reported.^24^ Thus, the regulation of p53 on autophagy is complicated and controversial, depending on the cell‐type and stress conditions.

p53 are frequently mutated in GBM, and the role and impact of mutant p53 in autophagy regulation is complex, context‐dependent and far from fully elucidated. In present study, we revealed hsa_circ_0072309 as a regulator of autophagy and TMZ sensitivity in p53 wild‐type GBM, but not in p53 mutant GBM. However, the p53 mutant cell lines (U251) used in our experiment is p53 R273h mutated. Whether other kind of p53 mutation affect the regulation is not clear. We will perform further studies to investigate this issue.

The regulation mechanism of autophagy in glioma is complicated. Some recent studies suggest that autophagy in glioma cells could be enhanced through other signaling pathways. Yang and his colleague claimed that the miR cluster MC‐let‐7a‐1 ~ let‐7d promotes glioma cell autophagy and apoptosis by repressing STAT3.^25^ It was reported synthetic cannabinoids induce autophagy and mitochondrial apoptotic pathways in GBM independently of deficiency in TP53 or PTEN.^26^ Olanzapine induced autophagy through suppression of NF‐κB activation in human glioma cells.^27^ Autophagy activated by silibinin contributes to glioma cell death via induction of oxidative stress‐mediated BNIP3‐dependent nuclear translocation of AIF.^28^

Taken together, our study reveals a key role of hsa_circ_0072309 in regulating autophagy and chemoresistance in p53 dependent manner. Low hsa_circ_0072309 expression predicts poor prognosis for glioma patients. The regulation of hsa_circ_0072309 on autophagy and TMZ sensitivity depends on the status of p53. Hsa_circ_0072309 promoted autophagy by p53 signaling pathway and enhanced sensitivity of glioblastoma to temozolomide (TMZ) in p53 wild‐type GBM, but not in p53 mutant GBM. Hsa_circ_0072309 inhibits p53 ubiquitination and increases the stability of p53 protein in the context of p53 wild‐type. MiR‐100 mediates hsa_circ_0072309 regulating p53. P53 inhibitor or autophagy inhibitor could reverse the effect of hsa_circ_0072309 on TMZ sensitivity in p53 wild‐type GBM. These findings demonstrated that hsa_circ_0072309 may be a potential and promising target in designing the treatment strategy for GBM.

## CONFLICT OF INTEREST

The authors declare that they have no conflict of interest.

## AUTHOR CONTRIBUTIONS

The first authors Fanen Yuan and Si Zhang contributed equally to this work. Fanen Yuan designed the experiments and wrote the article; Si Zhang and Qian Sun performed the experiments; Liguo Ye and Yang Xu finished data analysis; Gang Deng and Zhou Xu prepared the article, Shenqi Zhang and Baohui Liu did the investigation; Qianxue Chen administrated the whole study. All authors approved the final article.

## Supporting information

Figure S1

Figure S2

Figure S3

Figure S4

Figure S5

Figure S6

Figure S7

## Data Availability

The data that support the findings of this study are available from the corresponding author upon reasonable request.
