# Supplementary figures and images for: Hsa_circ_0072309 enhances autophagy and TMZ sensitivity in glioblastoma
Source: CNS Neurosci Ther. 2022 Feb 25;28(6):897–912. doi: 10.1111/cns.13821 (PMC9062556; doi:10.1111/cns.13821)

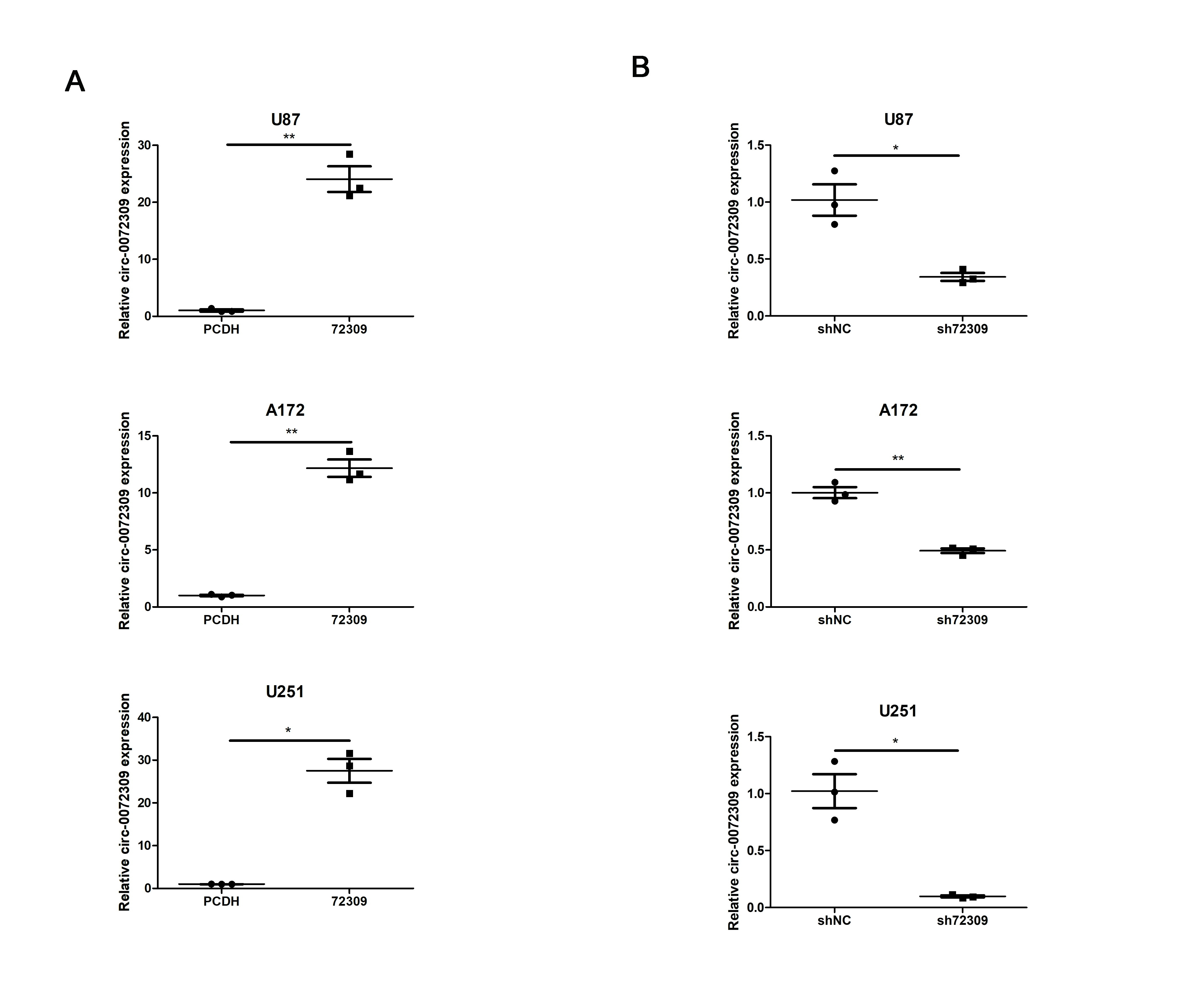

Supplement: Supplementary file 1 — Figure S1 [file CNS-28-897-s002.tif]

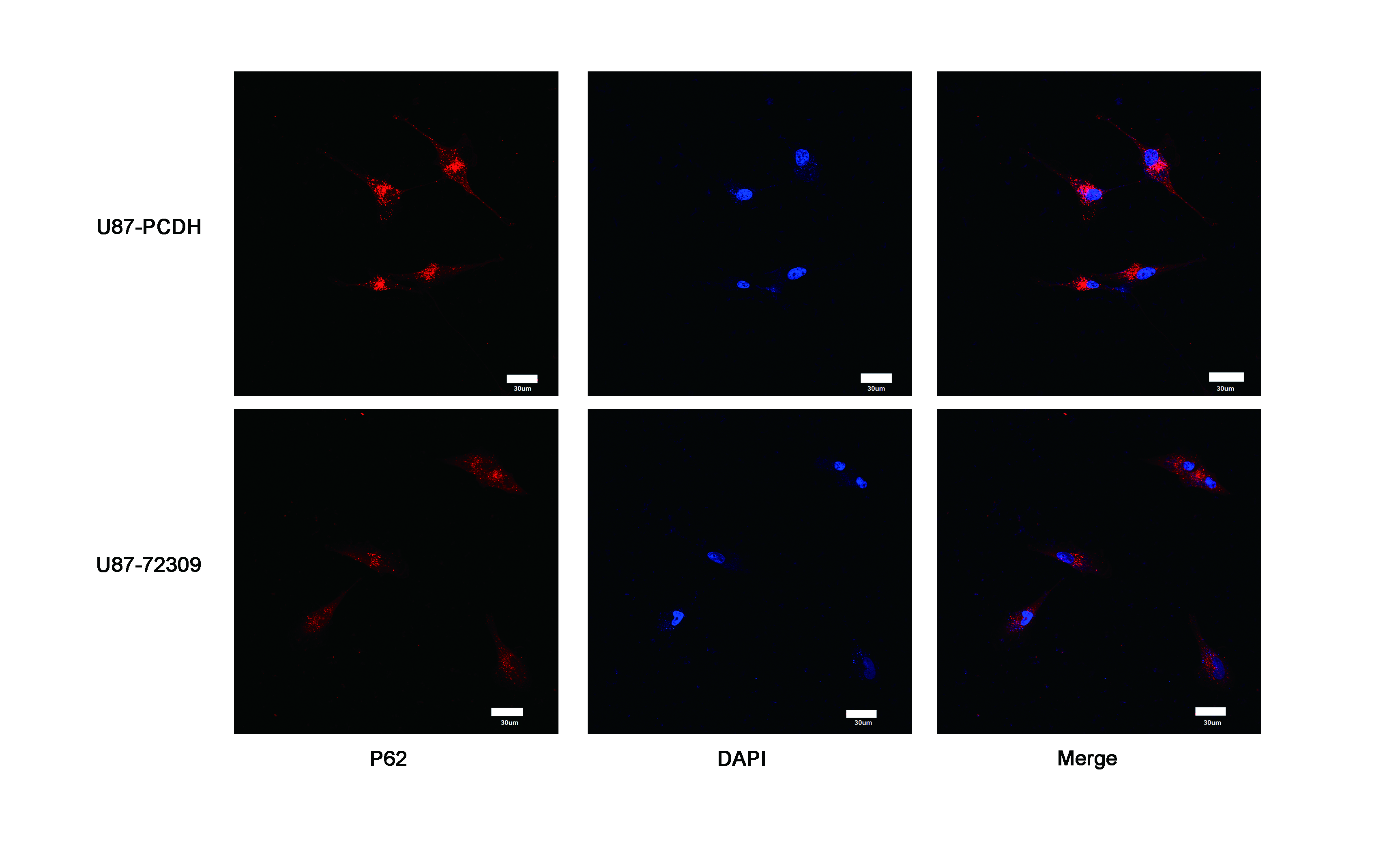

Supplement: Supplementary file 2 — Figure S2 [file CNS-28-897-s006.tif]

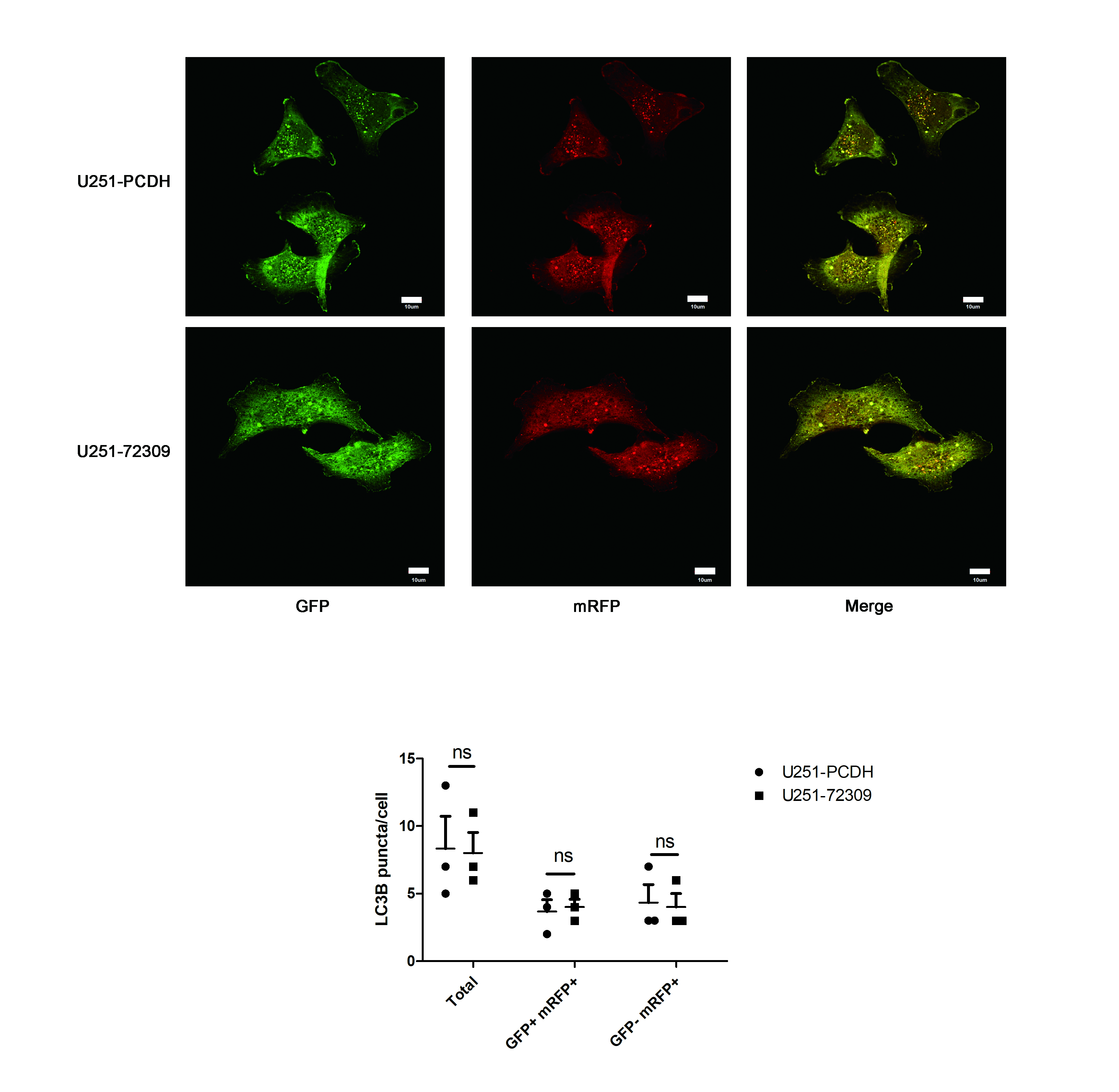

Supplement: Supplementary file 3 — Figure S3 [file CNS-28-897-s001.tif]

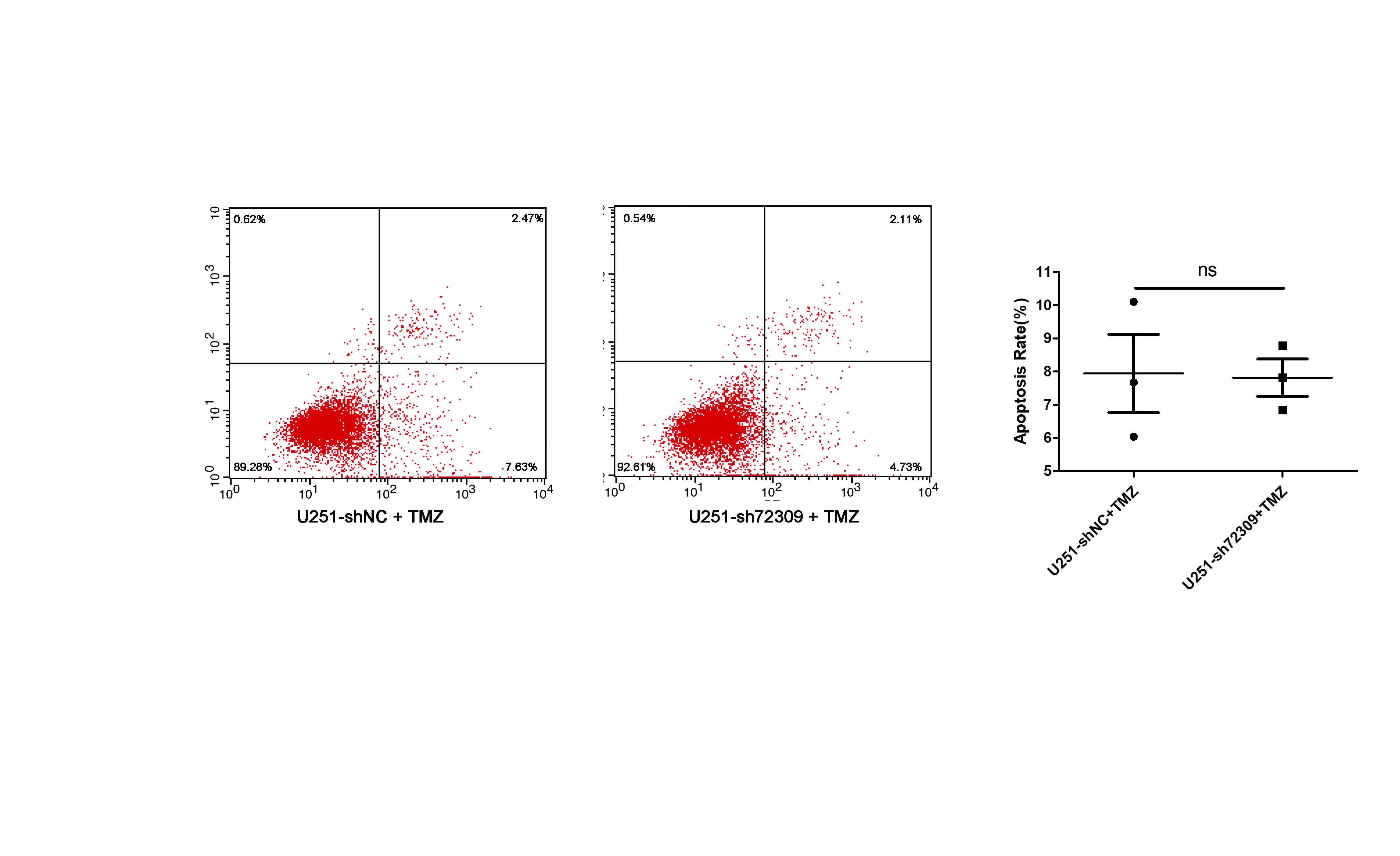

Supplement: Supplementary file 4 — Figure S4 [file CNS-28-897-s005.tif]

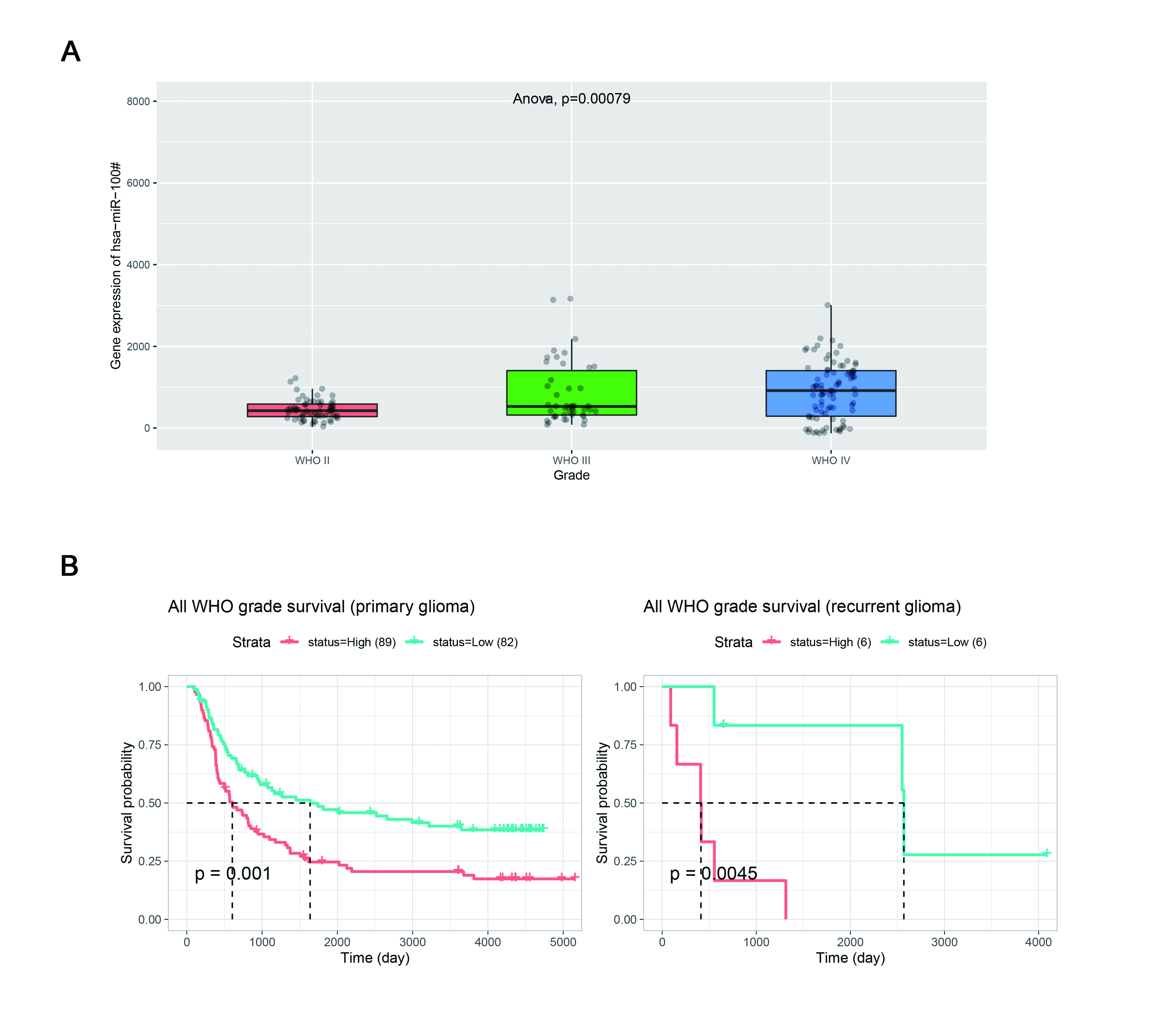

Supplement: Supplementary file 5 — Figure S5 [file CNS-28-897-s007.tif]

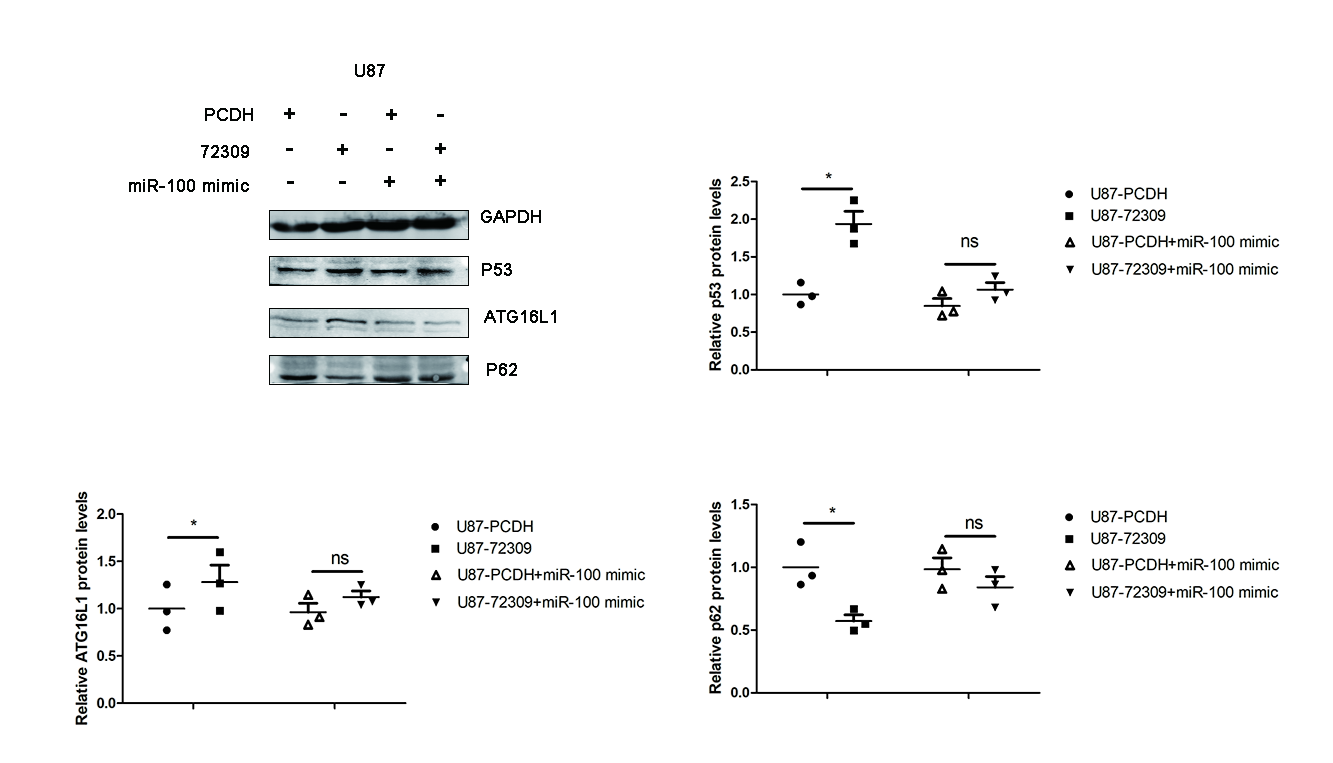

Supplement: Supplementary file 6 — Figure S6 [file CNS-28-897-s004.tif]

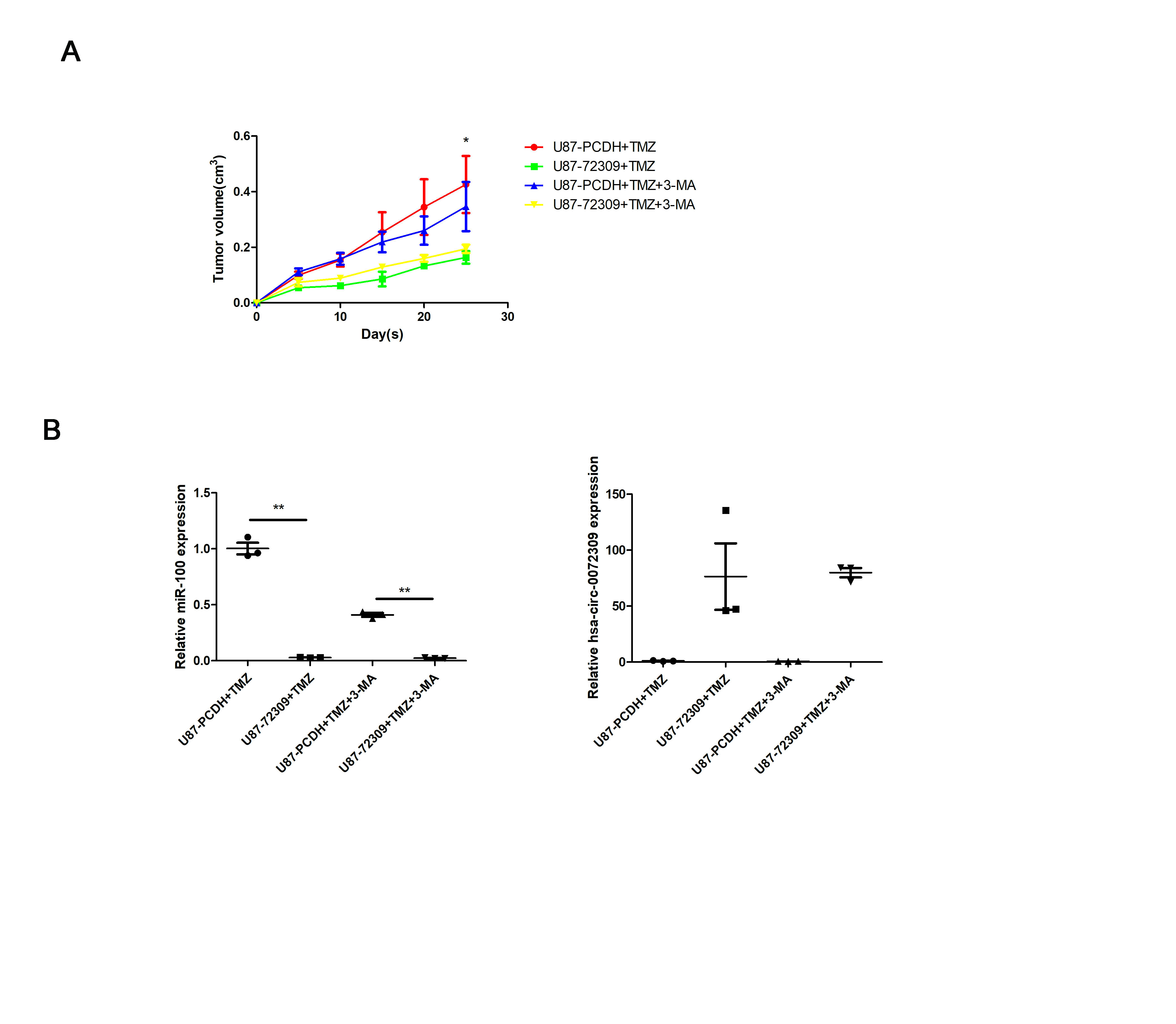

Supplement: Supplementary file 7 — Figure S7 [file CNS-28-897-s003.tif]
